# Impact of chronic heat stress on behavior, oxidative status and meat quality traits of fast-growing broiler chickens

**DOI:** 10.3389/fphys.2023.1242094

**Published:** 2023-09-12

**Authors:** Alice Cartoni Mancinelli, Giulia Baldi, Francesca Soglia, Simona Mattioli, Federico Sirri, Massimiliano Petracci, Cesare Castellini, Marco Zampiga

**Affiliations:** ^1^ Department of Agricultural, Food and Environmental Sciences, University of Perugia, Perugia, Italy; ^2^ Department of Agricultural and Food Sciences, Alma Mater Studiorum—University of Bologna, Bologna, Italy

**Keywords:** broiler chicken, heat stress, behavior, blood parameter, oxidation, meat quality

## Abstract

This research aimed to investigate, through a multifactorial approach, the relationship among some *in-vivo* parameters (i.e., behavior and blood traits) in broilers exposed to chronic HS, and their implications on proximate composition, technological properties, and oxidative stability of breast meat. A total of 300 Ross 308 male chickens were exposed, from 35 to 41 days of age, to either thermoneutral conditions (TNT group: 20°C; six replicates of 25 birds/each) or elevated ambient temperature (HS group: 24 h/d at 30°C; six replicates of 25 birds/each). In order to deal with thermal stress, HS chickens firstly varied the frequency of some behaviors that are normally expressed also in physiological conditions (i.e., increasing “drinking” and decreasing “feeding”) and then exhibited a behavioral pattern finalized at dissipating heat, primarily represented by “roosting,” “panting” and “elevating wings.” Such modifications become evident when the temperature reached 25°C, while the behavioral frequencies tended to stabilize at 27°C with no further substantial changes over the 6 days of thermal challenge. The multifactorial approach highlighted that these behavioral changes were associated with oxidative and inflammatory status as indicated by lower blood γ-tocopherol and higher carbonyls level (0.38 vs. 0.18 nmol/mL, and 2.39 vs. 7.19 nmol/mg proteins, respectively for TNT and HS; *p* < 0.001). HS affected breast meat quality by reducing the moisture:protein ratio (3.17 vs. 3.01, respectively for TNT and HS; *p* < 0.05) as well as the muscular acidification (ultimate pH = 5.81 vs. 6.00, respectively; *p* < 0.01), resulting in meat with higher holding capacity and tenderness. HS conditions reduced thiobarbituric acid reactive substances (TBARS) concentration in the breast meat while increased protein oxidation. Overall results evidenced a dynamic response of broiler chickens to HS exposure that induced behavioral and physiological modifications strictly linked to alterations of blood parameters and meat quality characteristics.

## 1 Introduction

The report of the International Panel on Climate Change (IPCC, 2022) depicted a concerning scenario in which, without a substantial reduction of greenhouse gas emissions, global warming could exceed the threshold established in the Paris Climate Agreement (i.e., maintain global warming under 2°C, preferably 1.5°C, respect to pre-industrial levels) within 2040. Albeit the negative effects of global warming on agriculture and livestock production were already evident in the past decades (FAO, 2016; Corwin, 2021), such forecasted increase of global temperature represents an additional challenge to satisfy, in an efficient and sustainable manner, the protein demand from a growing world population. Indeed, in tropical areas as well as in temperate climates during summer, the rise of environmental temperature can increase the risk for poultry and other livestock species to experience heat stress (**HS**), which takes place when animals are not capable of dissipating excess heat into the surrounding environment (Akbarian et al., 2016). Regardless of the duration (chronic vs. acute; Akbarian et al., 2016), HS is recognized as one of the most frequent and difficult to manage stressors that can occur during the rearing cycle (Aggarwal and Upadhyay, 2012), with negative implications on animal welfare, physiology and health as well as production sustainability, yields and product quality (Zaboli et al., 2019; Liu et al., 2020; Kumar et al., 2021; Brugaletta et al., 2022).

Broiler chickens are particularly susceptible to HS due to the presence of feathers, absence of sweat glands, high body mass-to-body surface area ratio, rapid metabolism and elevated core temperature (Syafwan et al., 2011). Decreased feed consumption as well as growth depression and physiological alterations (e.g., oxidative damage, fat deposition, protein catabolism, and histological modifications of the gastro-intestinal tract) were observed in broilers experiencing HS (Kikusato and Toyomizu, 2019; Lu et al., 2019; Mazzoni et al., 2022). Moreover, the artificial selection for high productive performance has modified energy partitioning in modern broilers favoring anabolic processes (e.g., efficient conversion of dietary energy into body growth; Zampiga et al., 2018), while negatively affecting their ability to deal with environmental stressors such as HS (Pawar et al., 2016).

In general, birds respond to high ambient temperatures by adjusting their behavior and physiological homeostasis in order to reduce body temperature (Lara and Rostagno, 2013). The identification of these changes is crucial to recognize and minimize the consequences of HS. It is important to underline that behavioral changes are the first bird response to thermal stress because of the lower energetic cost compared to other physiological adjustments (Branco et al., 2020). For instance, in HS conditions chickens spend more time resting, drinking, and panting and less time feeding, walking, and standing (Mack et al., 2013). The evaluation of the dynamic modifications of behavioral traits in response to the temperature increase can represent a valid information to rapidly identify thermal discomfort in broiler chickens, preserving animal welfare and health as well as productivity, with potential applications in the “precision livestock” context. On the other hand, the behavioral observations after a prolonged period of chronic HS can provide indications on the potential adaptive mechanisms adopted by the birds to cope with such conditions. In addition, only few studies have considered the overall relationships existing among animal behavior and oxidative balance, as well as their consequences on breast meat quality traits, in modern broilers exposed to chronic HS. Based on these considerations, the aim of this research was to investigate, through a multifactorial approach, the relationship among some *in-vivo* parameters (i.e., behavior and blood traits) in broilers exposed to chronic HS, and their implications on proximate composition, technological properties, and oxidative stability of breast meat.

## 2 Material and methods

### 2.1 Animal housing and environmental conditions

The present research is part of the experimental trial reported in Zampiga et al. (2021), where animal housing is described in detail. Briefly, the trial was conducted in the research facility of the University of Bologna (Italy) according to EU Regulation for the protection of meat-type chickens (European Commission, 2007), the protection of animals at the time of killing (European Commission, 2009), and the protection of animals used for scientific purposes (European Commission, 2010). The Ethical Committee of the University of Bologna authorized the experimental protocol (ID: 1031/2019).

For this research, two different rooms were used in order to rear the chickens under thermoneutral (**TNT**) or HS conditions. The rooms presented the same characteristics regarding artificial lighting and ventilation systems, and were equipped with six pens/room showing identical features. Each pen was 3.3 m^2^ and furnished with one circular pan feeder and five nipples. A total of 300 one-day-old Ross 308 male chickens were purchased and vaccinated at the hatchery (Marek, Newcastle, Gumboro, and coccidiosis), and then randomly allocated in either the TNT or HS room (25 chickens/pen). For the whole rearing cycle (0—41 days), the ambient temperature of the TNT room was settled consistently with the recommendations of the breeding company guidelines (i.e., placement: 30°C; 3 days: 28°C; 6 days: 27°C; 9 days: 26°C; 12 days: 25°C; 15 days: 24°C; 18 days: 23°C; 21 days: 22°C; 24 days: 21°C; 27–41 days: 20 °C). In the HS room, chickens were reared in thermoneutral conditions until 35 days of age, and then exposed to a temperature of 30°C for 24 h/d up to slaughter (41 days). Overall, the temperature increase in HS room was progressive, approximately 1°C–1.5°C per h on average. Such conditions (i.e., temperature, time of exposure, *etc.*) were chosen in order to simulate a chronic heat wave occurring the last week before slaughtering, so when modern broilers are particularly susceptible to high temperatures. Overall, the conditions adopted in this study were effective to induce a thermal stress response in the birds without significantly impacting livability (Zampiga et al., 2021). From 35 to 41 days, the temperature of both rooms was recorded through the use of three data loggers positioned at the beginning, middle, and end of each room. During the trial, the range of relative humidity was 40%–55% in both rooms. All birds received the same commercial basal diet (based on corn, wheat and soybean meal; mash form) according to a 3-phase feeding program: starter (0—14 days), grower (15—28 days) and finisher (29—41 days). For the entire period of trial, birds had free access to fresh water and feed, which were distributed *ad-libitum*.

### 2.2 Behavioral observations

Behavioral observations were conducted in three representative pens/room (i.e., beginning, middle, and end of each room) at 35, 36 and 41 days of bird age. Twelve chickens per pen (in total 36 birds/treatment) were randomly selected and individually labelled with stick spray on their back. To avoid any interference between the observer and the birds, the behavior was assessed with the Noldus Technology (Wageningen, the Netherlands), which consists of two software: Media Recorder and Observer XT. The Media Recorder allows to record a video with a camera (Basler, Wageningen, the Netherlands), which was then analyzed with the Observer XT using the instantaneous scanning sampling method (Altmann, 1974) following a pre-defined ethogram (Table 1). At 35 days, a video of 8 h length was recorded in the three pens of the HS room to evaluate the changes in bird behavioral pattern in response to the gradual increase of environmental temperature from 18°C to 30°C. In particular, as the temperature increase was progressive, videos were analyzed at every change in temperature (1°C) for 1 min. At 1 and 6 days of HS exposure (corresponding to 36 and 41 days of bird age), two videos a day, taken from 9 a.m. to 10 a.m. and from 3 p.m. to 4 p.m., were recorded in the same three pens of the TNT and HS room. According to Cartoni Mancinelli et al. (2020) and Cartoni Mancinelli et al. (2022), each video was analyzed by a single researcher with experience in poultry behavior and trained in the use of Noldus software, using the scan sampling method for a total of 60 observations of 5 s each *per* hour (*n* = 36 birds/group). All data are presented as the frequency of each behavior in the analyzed video (n./time).

**TABLE 1:** Ethogram used to analyze the behavior of broiler chickens raised in either thermoneutral (TNT) or chronic heat stress (HS) conditions (*n* = 36/group) from 35 to 41 days of age.

Category	Behavior	Description
STATIC	Roosting	Lying position, the ventral body region is in contact with the floor
	Resting	The body is in line with the ground, the head is erected and eyes opened. Only the feet are in contact with the floor
	Sleeping	The head is in a low posture (under the wing or on the bedding) and eyes closed
ACTIVE	Walking	Moving more than three steps
EAT	Feeding	Pecking inside the feeder
	Drinking	Pecking the drinker
HEAT	Panting	Showing fast, laboured breathing with an opened beak
	Elevating wings	Wings are spaced from the body

### 2.3 Blood parameters

As reported in Zampiga et al. (2021), 12 birds/group (2 birds/replication) were chosen at 41 days based on the average body weight of each experimental group (2,900 and 2,450 g, respectively for TNT and HS; *Δ* = ± 50 g for both groups). At slaughtering in a commercial plant (using electrical stunning as described below; European Commission, 2009), blood samples were obtained from the 12 selected broilers/group. Blood was collected in heparinized vacutainers and centrifuged (1,500 × *g* for 10 min at 4°C) to collect plasma, while serum was obtained by spontaneous separation in tubes kept 2 h at ambient temperature. Both plasma and serum samples were stored at −80°C until analyses. As reported in Mattioli et al. (2019), the plasma lipid peroxidation was determined through a spectrophotometer (Shimadzu Corporation UV-2550, Kyoto, Japan) set at 532 nm according to the absorbance of thiobarbituric acid reactive substances (**TBARS**) and a tetraethoxypropane calibration curve in sodium acetate buffer (pH = 3.5). Accordingly, the results were expressed as nmol of malondialdehyde (**MDA**)/mL of plasma. The method proposed by Dalle-Donne et al. (2003), based on the use 2,4-dinitrophenylhydrazine (**DNPH**) as reactive, was applied to determine the protein carbonyl groups. Furthermore, the serum carbonyl content (reported as nmol/mg of protein) was evaluated at 366 nm of absorbance using 22,000 M 1/cm as a molar absorption coefficient. Before that analysis, the serum was diluted to 1:40 with phosphate-buffered saline.

The concentrations of tocols (α-tocopherol and its isoforms γ and δ, and α-tocotrienol) and retinol were determined following the Schüep and Rettenmaier (1994) protocol. In detail, the plasma (0.2 mL) was mixed with 4 mL of an ethanol solution containing 0.06% butylated hydroxytoluene (**BHT**) and 1 mL of water. Water/potassium hydroxide (60%) was used to saponify the mixture at 70°C for 30 min, which was then extracted with hexane/ethyl acetate (9/1, v/v). After centrifugation, a volume of 2 mL of the supernatant was transferred into a glass tube, dried under N_2_, and re-suspended into 200 μL of acetonitrile. The pellet was re-extracted twice and the filtrate (50 μL) was injected into an HPLC (pump model Perkin Elmer series 200, equipped with an autosampler system, model AS 950-10, Jasco, Tokyo, Japan) on a Sinergy Hydro-RP column (4 μm, 4.6 × 100 mm; Phenomenex, Bologna, Italy) setting 2 mL/min as flow rate. The identification of the tocopherols and tocotrienols was done by means of a fluorescence detector (model Jasco, FP-1525) with excitation and emission wavelengths of 295 and 328 nm, respectively. External calibration curves, constructed with increasing quantities of pure standard solutions (Sigma-Aldrich, Bornem, Belgium) in ethanol, were used to quantify the related tocopherols. The same HPLC device, equipped with a UV-VIS spectrophotometer detector (Jasco UV2075 Plus) set at λ 325 nm, was used to assess the retinol. For the identification and quantification of retinol, the sample was compared with a pure commercial standard in chloroform (Extrasynthese, Genay, France; Sigma-Aldrich, Steinheim, Germany).

The Folch et al. (1957) method was adopted for serum lipid extraction, while the esterification was carried out following the methodology proposed by Christie (1982). The heneicosanoic acid methyl esters (Sigma Chemical Co.) were used as the internal standard for the trans-methylation procedure with recovery rates of 89% ± 4%. A Varian gas chromatograph (CP-3800), equipped with a flame ionization detector and a capillary column (100 m length × 0.25 mm × 0.2 μm film; Supelco, Bellefonte, PA, United States), was utilized for the analysis of the fatty acid composition (described in detail in Mattioli et al., 2021). The flow rate was 2 mL/min with helium as carrier gas and the split ratio was equal to 1:80. The oven temperatures were as follows: 40°C for 1 min, then 163°C for 10 min (rate of 2°C/min), 180°C for 7 min (rate of 1.5°C/min), 187°C for 2 min (rate of 2°C/min), and finally 230°C for 25 min with a rate of 3°C/min. The temperatures of the injector and detector were 270°C and 300°C, respectively. For each sample, the identification of the individual fatty acid methyl esters was carried out by comparing the peak retention times with those of the standard mixture (FAME Mix Supelco). The results were expressed as percentage of each individual fatty acid methyl ester on the total fatty acids methyl esters detected. Finally, the sum of the total saturated fatty acids (**SFA**), total monounsaturated fatty acids (**MUFA**), and total polyunsaturated fatty acids (**PUFA**) of the n-3 and n-6 series were calculated using the average amount of each fatty acid.

### 2.4 Meat quality traits

At 41 days, all birds were sent to a commercial processing plant (using the same, conventional truck; transport time ∼2 h) and then slaughtered according to routine practices. Both the groups were exposed to the same processing conditions. Birds were stunned by means of an electrified water-bath (200–220 mA, 1,500 Hz; European Commission, 2009) and killed through neck vessels severing, which was performed through an automatic device. After bleeding, carcasses were scalded (approximately 50°C for 210 s), mechanically plucked, eviscerated and processed (i.e., removal of head, neck, abdominal fat, and feet), and finally air-chilled to reach a core temperature of about 4°C–5°C. After 24 h, 15 carcasses per experimental group were individually weighed and dissected to obtain the *P. major* muscles. Breast yield (%) was calculated accordingly and then meat used for the analytical determinations. Breast meat proximate composition was evaluated following the procedures reported by the Association of Official Analytical Chemists (AOAC, 1990). Moisture and ash evaluation was performed in duplicate, whereas the total fat and crude protein content was determined by means of the Folch et al. (1957) and the Kjeldahl copper catalyst (AOAC, 1990) methods, respectively. As concerns the technological properties of breast meat, color (L* = lightness, a* = redness, and b* = yellowness; CIE, 1978) was measured in triplicate on the muscle ventral (bone side) surface at 24 h post-mortem by means of a reflectance colorimeter (Chroma Meter CR-400; Minolta Corp., Milan, Italy). The iodoacetate method (Jeacocke, 1977) was applied to evaluate ultimate pH (pHu) of breast muscles. Briefly, 2.5 g of meat were minced and then homogenized for 30 s at 13,500 rpm by means of an Ultra-Turrax T25 basic (IKA-Werke, Germany) in solution (25 mL, pH 7.0) of 5 mM sodium iodoacetate and 150 mM potassium chloride. Finally, a Jenway 3510 pH-meter (Jenway, Cole-Parmer, Staffordshire, United Kingdom), calibrated at pH 4.0 and 7.0, was used to assess homogenate pH. For drip loss analysis, meat samples weighing approximately 80 g (8 cm × 4 cm × 2 cm) were obtained from the cranial portion of each *Pectoralis major* muscle, weighed, and then stored in plastic boxes over sieved plastic racks for 48 h at 4°C ± 1°C. Then, the samples were weighed back after blotting the excess surface fluids and drip loss was expressed as percentage of weight lost during refrigerated storage. Each sample utilized for drip loss analysis was then packaged under vacuum and cooked in a water bath upon reaching 80°C in the inner core. Samples were then cooled down at room temperature and weighed to calculate cook loss. Finally, cooked subsamples (4 cm × 2 cm × 1 cm) were used for shear force analysis, which was assessed through a TA. HDi Heavy Duty texture analyzer (Stable Micro Systems Ltd., Godalming, Surrey, United Kingdom) equipped with a 25 kg loading cell and an Allo-Kramer shear probe. Shear force results were expressed as kilogram per gram of meat.

### 2.5 Oxidation markers, antioxidants content and fatty acid proportions in the breast muscle

All oxidative parameters and the fatty acid profile of the breast muscle were assessed in triplicate. The content of α, γ and δ-tocopherol, α and γ-tocotrienol, and retinol were determined using an HPLC system following the method proposed by Hewavitharana et al. (2004). Briefly, 2 g of sample were included into a solution containing 5 mL distilled water and 4 mL ethanol, which was then vortexed for 10 s. Four mL of hexane containing BHT (200 mg/L) were included into the solution that was mixed and centrifuged (8,000 × g for 10 min). Then, 3 mL of supernatant were dried by N_2_ stream and dissolved into 200 μL of acetonitrile. A total of 50 μL was injected into the HPLC equipment and analyzed as indicated for the plasma. The peroxidability index was computed according to the formula defined in the work of Arakawa and Sagai (1986): (% monoenoic × 0.025) + (% dienoic × 1) + (% trienoic × 2) + (% tetraenoic × 4) + (% pentaenoic × 6) + (% hexaenoic × 8). As described before, the Folch et al. (1957) method was applied to extract the meat lipid fraction for fatty acid analysis. The gas chromatograph conditions were the same adopted for the serum fatty acids evaluation. As for the meat oxidative profile, TBARS were analyzed following the procedure proposed by Bao and Ertbjerg (2015), whereas protein carbonylation level was determined through the DNPH-based method (Soglia et al., 2016).

### 2.6 Statistical analyses

The STATA software (StataCorp LP., United States) was used for the statistical analysis of data concerning the animal behavior, in which the bird was considered as the experimental unit. For these traits, two different aspects were evaluated: i) the bird behavioral changes in response to the increase of environmental temperature at 35 days (from 18°C to 30°C), and ii) the effect of the prolonged exposure to HS (1 and 6 days) on behavior. The first dataset was analyzed by means of a mixed model considering the effect of HS accounting for the repeated measures performed on the same broiler chicken at different times. In the second dataset, the effect of HS on behavior was tested by one-way ANOVA with the fixed effect of the ambient condition (TNT vs. HS) and the repeated effect of the bird. As no significant differences ascribable to the time of recording (AM or PM) and HS exposure (1 and 6 days) were found, these effects were not included in the statistical model and thus the environmental condition was the only experimental factor. Significance was designated at *p* < 0.05 and the Bonferroni *post hoc* test was used. Moreover, a multivariate analysis was performed (Principal Component Analysis; **PCA**) to simultaneously assess the global trend of behavior (roosting, resting, sleeping, feeding, drinking, panting and elevating wings) and blood parameters (carbonyls and γ-tocopherol concentration). Panting and elevating wings were considered as a unique variable in the PCA being typical behaviors associated to HS in chickens. To reduce the number of Principal Components (**PC**), only those with eigenvalues > 1 were retained. The one-way ANOVA option of the GLM procedure of SAS software (SAS Institute Inc., United States) was applied for the analysis of data concerning meat quality traits and oxidative profile. The main effect of temperature was tested (TN vs. HS) and the single breast and bird were considered as the experimental unit, respectively. The Tukey’s HSD test with a significant level of *p* <0.05 was selected for means separation.

## 3 Results

### 3.1 Behavioral observations

**FIGURE 1:**
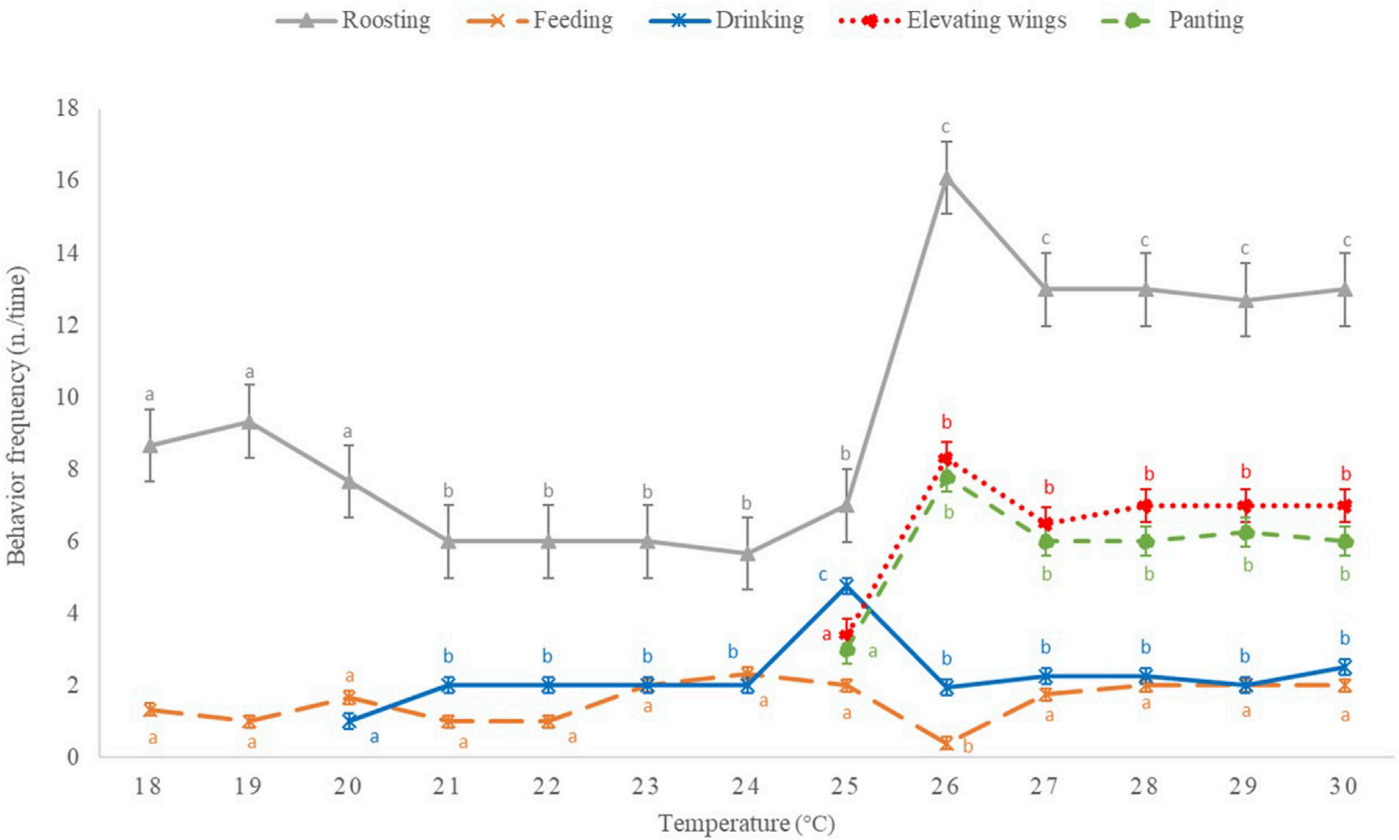
Frequency of the main behaviors expressed (n./time, time = minute) by broiler chickens during the 8 h of temperature increase from 18°C to 30°C (*n* = 36/group). Results are expressed as mean and standard error of the mean. ^a-c^: For each behavior, different letters indicate significant changes (*p* < 0.05) in its occurrence according to temperature variation.

Behavioral changes, both in terms of activity and frequency, were observed when the environmental temperature increased (Figure 1). In particular, when the temperature reached 25°C chickens exhibited the highest frequency of “drinking” (4.8 n./time). At this temperature, “panting” and “elevating wings” behaviors were observed for the first time. At the temperature of 26°C, the prevalent behaviors were “roosting” (16.1 n./time) followed by “elevating wings” and “panting” (8.3 and 7.8 n./time, respectively), while “feeding” was the only behavior not expressed by the chickens. Over 26°C, birds seemed to stabilize their behavior showing high frequency of “roosting”, “elevating wings” and “panting” followed by “drinking” and “feeding.”

**FIGURE 2:**
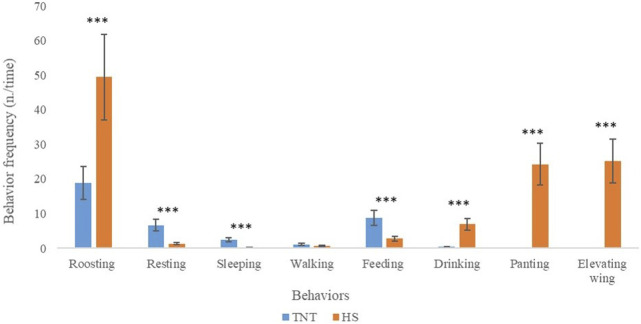
Behavioral frequencies (n./time, time = 2 h of length video/d) of broiler chickens raised in either thermoneutral or chronic heat stress conditions from 35 to 41 days of age (*n* = 36/group). Results are expressed as mean and standard error of the mean and represent the average of independent measurements carried out after 1 and 6 days of HS exposure. *** = *p* < 0.001.

The behavioral pattern of TNT and HS broilers exposed to chronic HS (1 and 6 days of exposure) are reported in Figure 2. Specifically, when compared to HS birds, TNT ones exhibited greater variability in the static behaviors with a higher frequency of “resting” and “sleeping” (6.64 vs. 1.32 and 2.48 vs. 0.16 n./time, respectively; *p* < 0.001). On the contrary, birds belonging to the HS group spent more time in “roosting” when compared to TNT (49.4 vs. 18.9 n./time, respectively; *p* <0.001). Concerning the eating behaviors, the HS group showed a higher frequency of “drinking” compared to the TNT one. On the contrary, TNT birds exhibited the highest frequency of “feeding.” The HS chickens also showed behaviors like “panting” and “elevating wings” that were not observed in the TNT ones.

### 3.2 Blood parameters

The blood oxidative parameters and the fatty acid profile of TNT and HS broilers were reported in Tables 2 and 3, respectively. The HS significantly affected the carbonyls content, which was 3-fold higher in HS birds than in TNT ones (7.19 vs. 2.39 nmol/mg proteins, respectively; *p* < 0.01). A different trend was observed for the tocopherol isoforms, with a higher δ-tocopherol concentration found in HS chickens (*p* < 0.01). On the contrary, the γ-tocopherol was higher in TNT than in HS group (*p* < 0.01), whereas no substantial difference was detected for α-tocopherol. As for the blood fatty acid profile, significant differences were only found on the Σ MUFA content, which was lower in HS birds (12.75% vs. 15.57%, respectively for TNT and HS, *p* < 0.001). This is mainly due to the lower concentration of oleic acid (C18:1 n-9) in HS birds (13.92% vs. 10.90%, respectively for TNT and HS; *p* < 0.001).

**TABLE 2:** Blood oxidative parameters of 41 days-old broiler chickens raised in either thermoneutral (TNT) or chronic heat stress (HS) conditions (*n* = 12/group) from 35 to 41 days of age.

	TNT	HS	SEM	*p*-value
Oxidative markers				
** **TBARS (nmol MDA/mL)	36.21	35.47	4.50	ns
Carbonyls (nmol/mg proteins)	2.39	7.19	2.50	***
Antioxidant content				
Retinol (nmol/mL)	6.32	5.89	1.00	ns
α-tocotrienol (nmol/mL)	0.005	0.110	0.012	ns
δ-tocopherol (nmol/mL)	0.15	0.31	0.11	***
γ-tocopherol (nmol/mL)	0.38	0.18	0.16	***
α-tocopherol (nmol/mL)	18.78	19.19	5.76	ns

***= *p* < 0.001; ns = not significant.

TBARS, thiobarbituric acid reactive substances.

MDA, malondialdehyde.

SEM, standard error of the mean.

**TABLE 3:** Blood fatty acids proportion (% of total fatty acids) in 41 days-old broiler chickens raised in either thermoneutral (TNT) or chronic heat stress (HS) conditions (*n* = 12/group) from 35 to 41 days of age.

	TNT	HS	SEM	*p-*value
C14:0	0.40	0.50	0.34	ns
C16:0	12.2	13.1	0.10	ns
C17:0	0.16	0.14	0.27	ns
C18:0	12.5	13.5	0.03	ns
C24:0	1.90	2.54	0.67	ns
ΣSFA	27.2	29.7	0.25	ns
C14:1	0.08	0.06	0.90	ns
C16:1	0.99	1.02	0.02	ns
C17:1	0.05	0.10	0.04	ns
C18:1 n-9	13.9	10.9	0.02	***
C24:1	0.47	0.64	0.92	ns
ΣMUFA	15.6	12.8	0.19	***
C18:2 n-6 [LA]	41.2	39.7	0.84	ns
C18:3 n-6 [GLA]	0.05	0.01	0.91	ns
C20:4 n-6 [AA]	6.77	7.27	0.01	ns
C22:2 n-6	0.24	0.12	0.34	ns
Σn-6	48.0	47.0	0.04	ns
C18:3 n-3 [ALA]	1.34	1.55	0.90	ns
C18:4 n-3	0.08	0.03	0.10	ns
C20:3 n-3	0.07	0.02	0.03	ns
C20:5 n-3 [EPA]	1.05	0.83	0.02	ns
C22:6 n-3 [DHA]	1.10	0.82	0.95	ns
Σn-3	3.65	3.26	0.08	ns
ΣPUFA	52.0	50.4	0.19	ns
n-6/n-3	13.3	15.3	0.89	ns
Others	5.30	6.90	1.02	ns

***= *p* < 0.001; ns = not significant.

SEM, standard error of the mean.

LA, linoleic acid; GLA, gamma-linolenic acid; AA, arachidonic acid; ALA, alpha-linolenic acid; EPA, eicosapentaenoic acid; DHA, docosahexaenoic acid.

### 3.3 Association among behavioral measures and blood parameters

**FIGURE 3:**
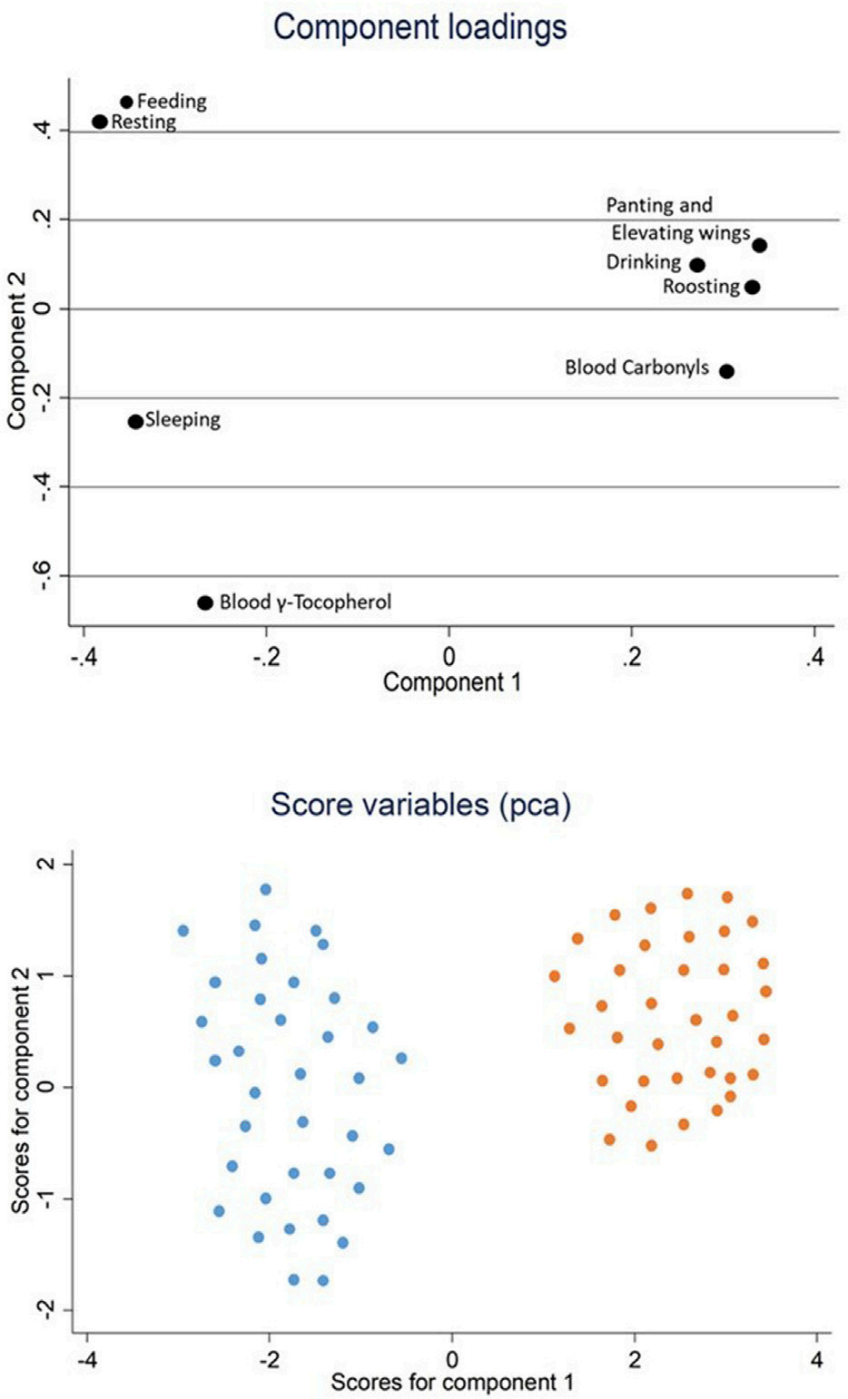
Plot of multivariate analysis related to the interaction between behavioral and blood parameters of broiler chickens raised in either thermoneutral (TNT; blu dots) or heat stress (HS; orange dots) conditions at 41 days of age (*n* = 36/group).

The plot of multivariate analysis (Table 4; Figure 3) showed the interaction of behavioral and blood parameters in TNT and HS chickens. The first two extracted PC with eigenvalues greater than 1.00 explained 77.8% of the total variance (Table 4). A positive value in the PC1 was observed for “roosting,” “drinking,” blood carbonyls, “panting and elevating wings.” Conversely, negative loadings were found for “feeding,” “resting,” “sleeping” and blood γ-tocopherol. In PC2, “roosting,” “feeding,” “resting”, “drinking”, “panting and elevating wings” showed positive value whereas “sleeping, blood carbonyls and blood γ-tocopherol exhibited a negative one. In Figure 3, the scores revealed a clear separation of the two experimental groups (TNT and HS). In particular, the HS birds were mostly discriminated by blood carbonyls, “drinking”, “roosting”, “panting and elevating wings”, whereas the TNT chickens were mainly characterized by “feeding”, “sleeping”, “resting” and blood γ-tocopherol.

**TABLE 4:** Eigenvalue, explained variance and loadings of the first two Principal Components (PC) of the multivariate analysis regarding the interaction between behavioral and blood parameters in TNT (thermoneutral) and HS (heat stress) chickens at 41 days of age.

Variable	PC1	PC2
Eigenvalue	5.24	4.98
Proportion	65.6	12.3
Cumulative	77.8	
Roosting	0.391	0.057
Feeding	−0.358	0.480
Resting	−0.381	0.435
Drinking	0.333	0.130
Sleeping	−0.338	−0.256
Panting and elevating wings	0.405	0.146
Blood carbonyls	0.347	−0.119
Blood γ-tocopherol	−0.247	−0.676

### 3.4 Meat proximate composition and quality traits

Broilers exposed to chronic HS conditions exhibited lower carcass weight (*p* <0.01) and breast yield (*p* <0.05) than those raised in the TNT environment (Table 5). Regarding the chemical composition of breast meat (Table 5), no remarkable difference was detected for the percentage of total lipid and ash between the TNT and HS groups. However, HS samples exhibited lower moisture and higher protein content when compared to the TNT ones (75.0% vs. 76.0%; *p* < 0.05, and 23.7% vs. 22.4%; *p* < 0.01, respectively). Consequently, a lower moisture:protein ratio was detected in breast meat from HS birds (3.01 vs. 3.17, respectively from HS and TNT; *p* < 0.05). HS substantially affected the main quality traits and technological properties of chicken breast meat (Table 5). Meat color was not affected by the environmental conditions, albeit lightness tended (*p* = 0.06) to be lower in HS samples which, on the other hand, exhibited higher pHu compared to the TNT ones (6.00 vs. 5.81, *p* < 0.01). Water holding capacity was found to be enhanced in HS fillets, as suggested by their lower drip and cooking losses (*p* < 0.05 and *p* < 0.001, respectively) when compared to TNT group that, in turn, exhibited higher shear force (*p* < 0.05).

**TABLE 5:** Chemical composition and meat quality traits of breast meat from broiler chickens (41 days-old) raised in either thermoneutral (TNT) or chronic heat stress (HS) conditions (n = 15/group) from 35 to 41 days of age.

	TNT	HS	SEM	*p*-value
Carcass weight (g)	2,031	1,753	50.8	**
Breast yield (%)	39.0	35.3	0.82	*
Moisture (%)	76.0	75.0	0.25	*
Crude protein (%)	22.4	23.7	0.29	**
Total lipid (%)	1.38	1.55	0.11	ns
Ash (%)	1.40	1.49	0.06	ns
Moisture:protein ratio	3.17	3.01	0.04	*
Lightness (L*)	56.9	54.8	0.56	0.06
Redness (a*)	1.12	1.14	0.11	ns
Yellowness (b*)	6.05	5.94	0.21	ns
pH_u_	5.81	6.00	0.03	**
Drip loss (%)	1.61	1.19	0.08	*
Cooking loss (%)	22.8	15.3	0.90	***
Shear force (kg/g)	3.11	2.71	0.10	*

*= *p* < 0.05; ** = *p* <0.01; *** = *p* <0.001. ns = not significant.

SEM, standard error of the mean.

### 3.5 Oxidation markers, antioxidants content and fatty acid proportions in the breast muscle

The results regarding the effects of HS on meat oxidative profile and antioxidant content are reported in Table 6. If compared to TNT, meat samples belonging to HS group exhibited significantly lower TBARS level and higher carbonyls content (5.23 vs. 4.70 mg MDA/kg of meat; *p* < 0.01, and 1.52 vs. 1.77 nmol/mg protein; *p* < 0.05, respectively). However, no significant difference in breast antioxidants content (Table 6) and fatty acid proportion (Table 7) was found between the TNT and HS birds.

**TABLE 6:** Meat oxidative profile and antioxidants content in 41-d-old broiler chickens raised in either thermoneutral (TNT) or chronic heat stress (HS) conditions (*n* = 12/group) from 35 to 41 days of age.

	TNT	HS	SEM	*p*-value
Oxidative markers				
** **TBARS (mg MDA/kg of meat)	5.23	4.70	0.10	**
Carbonyls (nmol/mg of protein)	1.52	1.77	0.05	*
Antioxidant content				
Retinol (µg/g)	4.79	3.11	0.57	ns
γ-tocotrienol (µg/g)	0.12	0.09	0.02	ns
α-tocotrienol (µg/g)	0.01	0.01	0.01	ns
δ-tocopherol (µg/g)	0.02	0.02	0.01	ns
γ-tocopherol (µg/g)	0.10	0.07	0.01	ns
α-tocopherol (µg/g)	5.10	3.76	0.61	ns
Peroxidability index	15.0	18.1	2.04	ns

*= *p* < 0.05; ** = *p* <0.01; ns = not significant.

SEM, standard error of the mean.

**TABLE 7:** Fatty acid proportion (% of total fatty acids) in the breast muscle of 41 days-old broiler chickens raised in either thermoneutral (TNT) or chronic heat stress (HS) conditions (N = 12/group) from 35 to 41 days of age.

	TN	HS	SEM	*p-*value
C14:0	0.33	0.31	0.16	ns
C16:0	18.6	17.8	0.51	ns
C17:0	0.25	0.27	0.02	ns
C18:0	8.49	8.62	0.45	ns
C24:0	0.13	0.16	0.14	ns
ΣSFA	27.8	27.2	0.62	ns
C14:1	0.06	0.06	0.01	ns
C16:1	2.40	2.46	0.20	ns
C17:1	0.14	0.17	0.01	ns
C18:1 n-9	26.6	26.0	0.84	ns
C18:1 *cis11*	2.23	2.13	0.06	ns
C24:1	0.01	0.01	0.01	ns
ΣMUFA	31.5	30.9	0.97	ns
C18:2 n-6 [LA]	28.6	28.3	0.43	ns
C18:3 n-6 [GLA]	0.26	0.29	0.02	ns
C20:4 n-6 [AA]	4.22	4.81	0.51	ns
C22:4 n-6	0.05	0.03	0.32	ns
C22:5 n-6	0.06	0.08	0.05	ns
Σn-6	33.2	33.5	0.48	ns
C18:3 n-3 [ALA]	2.41	2.57	0.09	ns
C18:4 n-3	0.15	0.05	0.05	ns
C20:3 n-3	0.13	0.10	0.02	ns
C20:5 n-3 [EPA]	1.07	1.24	0.14	ns
C22:5 n-3 [DPA]	0.86	1.01	0.11	ns
C22:6 n-3 [DHA]	0.39	0.54	0.06	ns
Σn-3	5.03	5.53	0.28	ns
ΣPUFA	39.1	39.9	0.80	ns
n-6/n-3	6.86	6.19	0.27	ns
Others	1.69	1.89	0.08	ns

ns = not significant.

SEM, standard error of the mean.

LA, linoleic acid; GLA, gamma-linolenic acid; AA, arachidonic acid; ALA, alpha-linolenic acid; EPA, eicosapentaenoic acid; DHA, docosahexaenoic acid.

## 4 Discussion

Raising broiler chickens in chronic HS conditions led to several changes in behavioral, oxidative and meat quality traits. Behavioral parameters could be considered useful tools for assessing the welfare state of an animal. It is well known that, when exposed to a stressful stimulus, animals tend to modify their behavior to adapt themselves to the new condition (Mack et al., 2013). In this study, we found that these behavioral changes could be divided into two different types: 1) frequency modification of normally expressed behaviors, and 2) activation of behaviors not previously expressed. As expected, during the 8 h in which the environmental temperature was increased from 18°C to 30°C (35 days of bird age), a higher frequency of “drinking” behavior was exhibited by the chickens once a temperature of 25°C was reached. However, such activity was not enough to counteract the progressive increase of temperature, so that new distinctive behaviors (“panting” and “elevating wings”) were expressed by the birds. Indeed, an increased frequency of “panting and elevating wings” was revealed starting from a temperature of 25°C. It should be noted that this behavioral pattern was observed only in HS broilers, indicating that chickens raised under heat stress tend to increase their body surface while exposing featherless areas in order to dissipate heat with the “elevating wings” and by increasing the evaporation through “panting” (Elshafaei et al., 2021). Moreover, a drastic decrease of “feeding” behavior was observed when the environmental temperature approached 26°C. The behavior pattern seems to stabilize at 27°C and it is characterized by a high frequency of “roosting.” The 8-h monitoring confirmed that behavioral changes are closely related to the rising of temperatures. In particular, two temperature thresholds were identified: the first at 25°C, where the animals activate new behaviors to dissipate heat, and the second one at 27°C when animals tend to stabilize their behavior pattern, exhibiting an increased frequency of static behaviors that are typical of heat-stressed chickens. This aspect was also confirmed by the evaluation of behavior pattern of birds undergoing a prolonged exposure to HS. Indeed, in our study no significant differences were observed in the behavior of chickens after 1 or 6 days of thermal stress, indicating that the animals tend to adapt their behavior to the new condition without substantial changes over time. In fact, although the “roosting” behavior is peculiar of broiler chickens (Bizeray et al., 2002), the HS condition drastically increased its frequency. It is reported that “roosting” facilitates heat exchange with the litter that generally presents a lower temperature than the bird (Branco et al., 2020). The higher frequency of “roosting” in HS chickens was associated with a reduced expression of “feeding” behavior. This outcome corroborates the results shown in our companion paper (Zampiga et al., 2021), in which a 33% reduction of feed intake was reported for HS birds compared to TNT ones. Similarly, Talebi et al. (2022) showed that both broilers and laying hens spend less time eating when exposed to high environmental temperatures. Moreover, in the study of Attia et al. (2018), HS negatively affected feed intake and, in turn, weight gain and feed efficiency. In line with our results, Branco et al. (2020) showed that, through the use of a Generalized Sequential Patterns (GSP) algorithm, chickens exposed to HS remained inactive thus evidencing that the environmental conditions were not favorable. Moreover, the ambient temperature was reported to be positively correlated with the “drinking” and negatively with the “running” (Bell and Weaver, 2002). Active behaviors such as “walking” or “eating” are known to cause an increase in body temperature (Marìa et al., 2004). Hence, the decreased activity of HS broilers can be considered as an adaptive response to reduce heat generation. It is important to underline that the longer is the time of thermal discomfort, the greater are the changes in the physiological status, productive performance and meat quality characteristics (Xing et al., 2019).

The multifactorial approach adopted in the present study allowed to point out the complex relationship existing among the *in-vivo* aspects considered (i.e., behavior and blood oxidative status), yet demonstrating a clear distinction between the TNT and HS group. Specifically, HS chickens were characterized by “drinking,” “roosting,” “panting” and “elevating wings” behaviors associated with carbonyls blood concentration. On the contrary, TNT birds were identified by “feeding,” “resting” and “sleeping” behaviors associated with γ-tocopherol blood level. The physiological and behavioral response of broilers chickens is a complex and dynamic outcome aimed at achieving homeostasis and adapting them to the new environmental conditions. The results of this study highlighted such dynamic trend. In fact, the multifactorial approach could be defined as an “image capture” of the *in-vivo* mechanisms adopted by the animals to overcome the HS and thus to return to their physiological homeostasis status. In this study, HS broilers showed higher blood carbonyls and lower γ-tocopherol concentration compared to thermoneutral birds. Many authors (Sykes and Fataftah, 1986; Ali et al., 2010) indicated that the blood carbonyls level can be considered as a biomarker of oxidative stress and protein peroxidation, whereas the γ-tocopherol exerts many beneficial functions especially as anti-inflammatory agent. So that, through such approach it is possible to speculate that the behaviors mostly characterizing the HS chickens (namely, “panting and elevating wings”) are strictly related to an inflammatory and oxidative status induced by the thermal challenge. The presence of inflammatory processes in HS birds is also confirmed by the higher heterophil-to-lymphocyte ratio detected in birds belonging to this experimental group (Zampiga et al., 2021). During the inflammatory process, the two isoforms of the cyclooxygenase enzymes (COX-1 and COX-2) convert the arachidonic acid into various prostanoids such as prostaglandins (PGs) (Verma et al., 2021), whose biosynthesis plays a key role in both the development and the propagation of the inflammatory signals (Ricciotti and FitzGerald, 2011). The γ-tocopherol is able to limit the synthesis of the inflammation mediator Prostaglandin E2 (PGE2) by inhibiting the enzyme COX-2 (Jiang et al., 2000). The prolonged exposure to chronic HS condition induces several metabolic alterations in broiler chickens. In fact, in normal physiological conditions, an energy unbalanced status can be restored through the mobilization of body fat (Lu et al., 2017). On the contrary, fat mobilization is suppressed under HS and the energy deficit, induced by the reduction of feed intake, enhances glucose metabolism (Akşit et al., 2006; Lu et al., 2017). Accordingly, a significantly lower MUFA content was detected in the blood of HS birds compared to TNT, which could be ascribed to a decreased synthesis of these compounds as a consequence of reduced feed intake. Moreover, TNT and HS birds exhibited the same fatty acid profile in both blood and meat, likely suggesting that no substantial variations occurred in terms of lipid mobilization between control and stressed birds.

**FIGURE 4:**
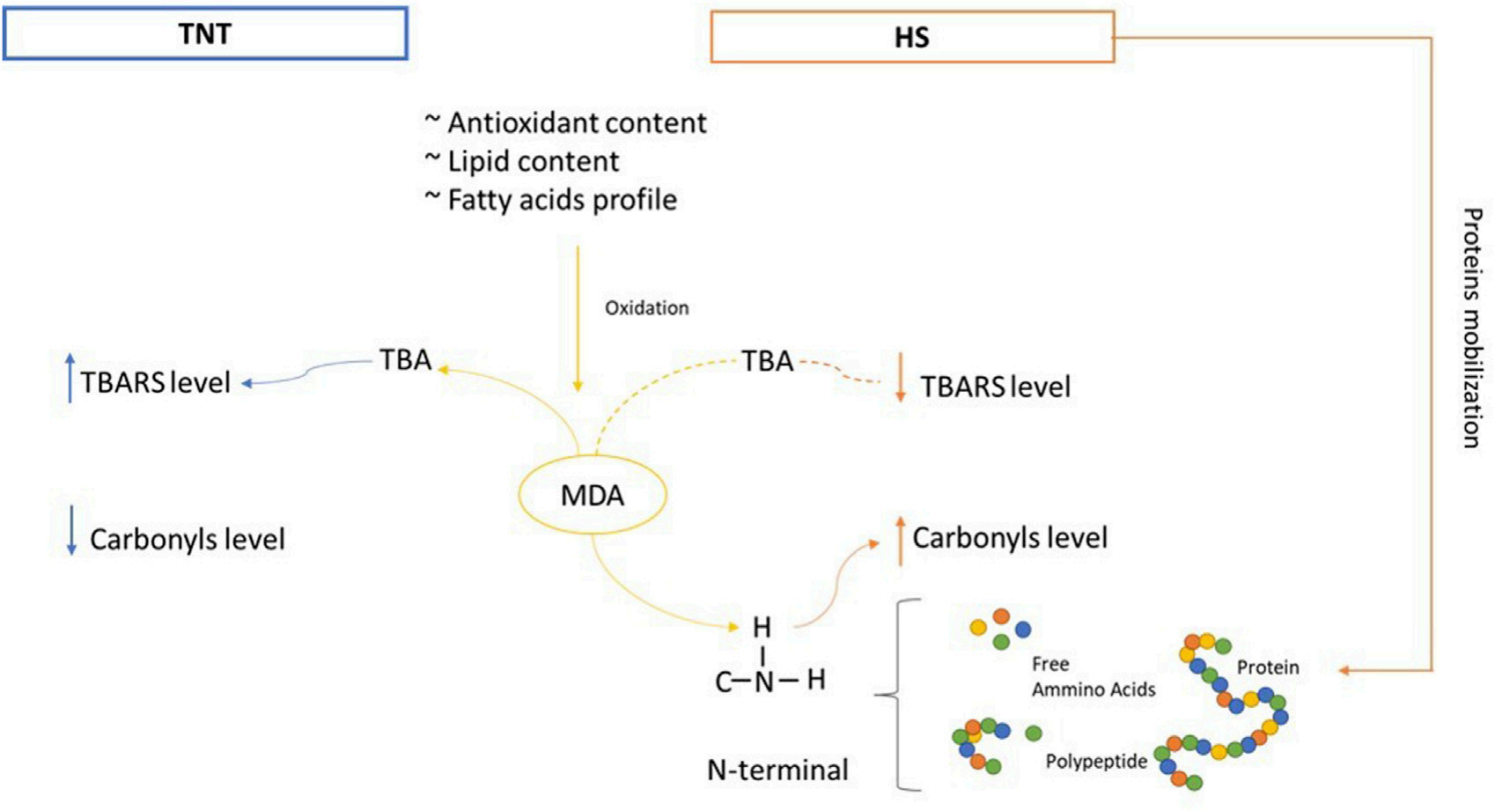
Hypothetical oxidative mechanism occurring in meat of broiler chickens raised in thermoneutral (TNT) or chronic heat stress (HS) conditions from 35 to 41 days of age. Antioxidants and lipids content as well as fatty acids profile are similar in the two groups. In both TNT and HS conditions, lipid oxidation occurs to the same extent, with the production of MDA (malondialdehyde). In TNT, MDA reacts with the TBA (thiobarbituric acid) to detect the TBARS level (thiobarbituric acid reactive substances). In HS, MDA reacts with the N-terminal group of free amino acids and polypeptides derived from protein mobilization. Carbonyls level increase while the reaction between MDA and TBA occurs to a lesser extent, thus resulting in a reduced TBARS detection.

Regarding the meat oxidative status, HS broilers showed higher carbonyls coupled with lower TBARS content. The latter outcome was rather unexpected considering that fatty acids profile and antioxidants concentration did not exhibit significant variations in response to the environmental conditions. HS has been associated with increased muscle protein degradation and catabolism (Lu et al., 2018) with consequent changes in amino acids metabolism (Tabiri et al., 2002; Ma et al., 2018). Thus, it has been hypothesized that HS may promote protein degradation in the muscle, thereby increasing the concentration of some amino acids in the blood (Rhoads et al., 2011; Zampiga et al., 2021). Some amino acids containing nucleophilic groups (e.g., histidine, cysteine, and lysine) can be indirectly carbonylated by binding with non-protein reactive carbonyl species, such as MDA, thus resulting in an MDA concentration-dependent increase of the protein carbonyl content (Wu et al., 2009). Considering that such modifications occur on the N-terminal of the peptides (Butterfield and Stadtman, 1997; Zhao et al., 2012), it could be supposed that the higher mobilization of proteins in response to the thermal challenge, which is supported by our previous investigation (Zampiga et al., 2021), might have increased the number of N-terminal groups available for the reaction with MDA. This phenomenon may ultimately result in a greater carbonylation level and reduced amount of MDA detectable through TBA-spectrophotometric assay (Figure 4). However, further insights are necessary to confirm such hypothesis.

It is widely recognized that HS is one of the most critical environmental factors affecting broiler growth performance due to the reduction in both feed intake and efficiency (Shakeri et al., 2020; Shakeri and Le, 2022). Within this context, broilers exposed to chronic HS exhibited a remarkable depression of both carcass weight and breast yield (−14% and −10%, respectively), which is consistent with the reduced “eating” behavior observed in the HS group and also with the lower final body weight and feed intake previously reported in Zampiga et al. (2021). Concerning meat proximate composition, breast muscles belonging to HS group were characterized by lower moisture and higher protein content. The lower amount of water found in HS group might be associated with an increased evapotranspiration that could result in dehydration and altered body water homeostasis, which can occur in heat-stressed birds especially when relative humidity is low (Yahav et al., 1995). On the other hand, the higher crude protein level detected in the breast muscles of HS group is rather counterintuitive considering the reduced feed intake and the increased catabolic processes associated with the thermal challenge. Possibly, such increase could indirectly result from a “concentration effect” determined by the lower water content rather than a greater *in-vivo* protein synthesis. Moreover, the reduced moisture:protein ratio found in the HS group suggests that a significantly lower quantity of water per amount of protein is present in the meat of HS birds, further supporting the hypothesis of a potential dehydration effect. In the EU legislation, the acceptable moisture:protein ratio for broiler breast meat is 3.19 ± 0.12 (range: 3.07–3.31) (Dias et al., 2020). Therefore, the average value detected in HS birds is out of range with possible relevant commercial issues for both internal EU market and exporters Countries.

HS can also alter muscular metabolism *in-vivo*, inducing several well-known final meat quality alterations whose extent eventually depends on both the duration and the magnitude of the stress (Wang et al., 2017; Gonzalez-Rivas et al., 2020). As for meat technological properties, it is widely accepted that exposing animals to acute HS immediately before slaughter accelerates muscle glycogenolysis and produces a rapid drop of muscular pH resulting in pale, soft and exudative meat, a condition responsible for impaired meat technological quality that can be frequently observed in poultry (Van Laack et al., 2000; Zampiga et al., 2020), pigs (Bowker et al., 2000), and cattle (Kim et al., 2014). By contrast, studies carried out on ruminants (Kadim et al., 2008) and pigs (D’Souza et al., 1998) disclosed that the exposure to chronic HS causes a reduction of muscular glycogen reserves and a feeble lactic acid production, thus resulting in higher pHu, darker color and greater water holding capacity of meat, i.e., common traits of dark, firm, and dry (**DFD**) meat. In poultry, the development of a DFD-like meat condition is more frequent in chickens kept in cold rather than heat stress conditions during pre-slaughter operations (Leishman et al., 2021), even though a prolonged exposure to high environmental temperatures can generate breast meat with high ultimate pH likely because of the stress-induced reduction of muscle glycogen reserves (Dai et al., 2012; Imik et al., 2012; Zeferino et al., 2016). Gregory (2010) indicated that extreme heat can provoke an adrenergic stress response with consequent increase of peripheral vasodilatation and muscle glycogenolysis, potentially resulting in meat with high pHu and darker color in case of protracted stress. In our study, muscles belonging to HS group exhibited a reduced extent of acidification as suggested by the higher pHu. Lower glucose levels *in-vivo* were found in the breast muscle of HS broilers by Zampiga et al. (2021), who hypothesized a boost of carbohydrate utilization in the glycolytic pathway of heat-stressed animals. On the other hand, the significant reduction of feed intake induced by the thermal challenge in HS broilers (−33% compared to TNT; Zampiga et al., 2021) could have also played a role on muscle glycolytic potential and thus on its acidification capacity. The higher pHu (6.00) found in HS samples, being far from the isoelectric point of myofibrillar proteins, likely enhanced their ability to retain constitutional water by increasing their net negative charge (Schreurs, 2000; Warriss, 2000). Accordingly, HS fillets exhibited an improved water holding capacity, as suggested by the remarkably lower drip and cooking losses (−26.1% and −33.0%, respectively) if compared to TNT ones. Moreover, shear force assessed on cooked meat was found to be lower in HS group, thus corroborating the inter-relationship between water holding capacity and meat tenderness, which establishes that a higher moisture content retained within the muscle results in a greater tenderness of meat (Hughes et al., 2014). Overall, it might be hypothesized that exposing birds to chronic HS during the last week before slaughtering could have affected muscle glycolytic potential and its acidification pattern, with consequences on color, water holding capacity and tenderness of breast meat. Although further insights are necessary to elucidate these dynamics, the depletion of muscle glycogen reserves *in-vivo* could be the result of the prolonged stress and its effects on metabolic traits and feed consumption. As mentioned above, the reported data were obtained in chronic HS performed to simulate the environmental conditions that could be experienced by the birds during an extreme heat wave in tropical regions or in temperate climates during summer. It is important to underline that the discordant results available in the literature could be related to the different heat stress conditions, including type (e.g., constant vs. cyclic), duration (e.g., days to weeks), and intensity (e.g., 28°C–34°C), which could remarkably affect the variables studied herein (Azad et al., 2010; Lu et al., 2019; Zaboli et al., 2019).

In conclusion, the present study highlights the changes in behavior, blood parameters, oxidative status and meat quality in broiler chickens subjected to chronic HS conditions. It was possible to identify a dynamic behavioral response of the animals to the rise of the environmental temperature, initially consisting in modifications of the frequency of some behaviors also expressed in thermoneutral conditions (i.e., increase of “drinking” and decrease of “feeding”), and then in the manifestation of behaviors aimed at dissipating heat, such as “panting” and “elevating wings.” Such modifications become evident when the temperature reached 25°C, while the behavioral frequencies tended to stabilize at 27°C with no further substantial changes over the 6 days of HS. The new behavioral patterns exhibited by HS chickens were linked to alteration of the blood parameters suggesting the presence of an oxidative (protein-induced) and inflammatory state. Chronic HS also affected the final meat quality by reducing muscular acidification, which led to abnormal meat water holding capacity and tenderness. Surprisingly, muscle TBARS concentration was lower in HS birds although protein oxidation occurred to a greater extent possibly due to an increased protein mobilization in response to the thermal challenge. However, further research is needed to better investigate this aspect.

## Data Availability

The raw data supporting the conclusions of this article will be made available by the authors, without undue reservation.

## Abbreviations

BHT, butylated hydroxytoluene; DFD, dark, firm, and dry; DNPH, dinitrophenylhydrazine; HS, heat stress; MUFA, monounsaturated fatty acids; PCA, principal component analysis; PC, principal component; PUFA, polyunsaturated fatty acids; SFA, saturated fatty acids; TBARS, thiobarbituric acid reactive substances; TNT, thermoneutral.

## References

[B1] Aggarwal A. Upadhyay R. (2012). Heat stress and animal productivity. New Delhi, India: Springer. 10.1007/978-81-322-0879-2

[B2] Akbarian A. Michiels J. Degroote J. Majdeddin M. Golian A. De Smet S. (2016). Association between heat stress and oxidative stress in poultry; mitochondrial dysfunction and dietary interventions with phytochemicals. J. Anim. Sci. Biotechnol. 7, 37–14. 10.1186/s40104-016-0097-5 27354915 PMC4924307

[B3] Akşit M. Yalcin S. Özkan S. E. Z. E. N. Metin K. Özdemir D. (2006). Effects of temperature during rearing and crating on stress parameters and meat quality of broilers. Poult. Sci. 85, 1867–1874. 10.1093/ps/85.11.1867 17032815

[B4] Ali M. N. Qota E. M. A. Hassan R. A. (2010). Recovery from adverse effects of heat stress on slow-growing chicks using natural antioxidants without or with sulphate. Int. J. Poult. Sci. 9, 109–117. 10.3923/ijps.2010.109.117

[B5] Altmann J. (1974). Observational study of behavior: sampling methods. Behaviour 49, 227–267. 10.1163/156853974X00534 4597405

[B6] Aoac (1990). Official methods of analysis. 15th ed. Washington USA: Association of Official Analytical Chemists.

[B81] Arakawa K. Sagai M. (1986). Species differences in lipid peroxide levels in lung tissue and investigation of their determining factors. Lipids 21, 769–775. 10.1007/BF02535410 3821392

[B7] Attia Y. A. Al-Harthi M. A. Elnaggar A. Sh. (2018). Productive, physiological and immunological responses of two broiler strains fed different dietary regimens and exposed to heat stress. Ital. J. Anim. Sci. 17, 686–697. 10.1080/1828051X.2017.1416961

[B8] Azad M. A. K. Kikusato M. Maekawa T. Shirakawa H. Toyomizu M. (2010). Metabolic characteristics and oxidative damage to skeletal muscle in broiler chickens exposed to chronic heat stress. Comp. Biochem. Physiol. A Mol. Integr. Physiol. 155, 401–406. 10.1016/j.cbpa.2009.12.011 20036750

[B9] Bao Y. Ertbjerg P. (2015). Relationship between oxygen concentration, shear force and protein oxidation in modified atmosphere packaged pork. Meat Sci. 110, 174–179. 10.1016/j.meatsci.2015.07.022 26241463

[B10] Bell D. D. Weaver W. D. (2002). Commercial chicken meat and egg production. 5th ed. Boston: Springer Science and Business Media LLC.

[B11] Bizeray D. Estevez I. Leterrier C. Faure J. M. (2002). Effects of increasing environmental complexity on the physical activity of broiler chickens. Appl. Anim. Behav. Sci. 79, 27–41. 10.1016/S0168-1591(02)00083-7

[B12] Bowker B. C. Grant A. L. Forrest J. C. Gerrard D. E. (2000). Muscle metabolism and PSE pork. J. Anim. Sci. 79, 1–8. 10.2527/jas.00.079ES1001c

[B13] Branco T. Moura D. J. Nääs I. A. Oliveira S. R. (2020). Detection of broiler heat stress by using the generalised sequential pattern algorithm. Biosyst. Eng. 199, 121–126. 10.1016/j.biosystemseng.2019.10.012

[B14] Brugaletta G. Teyssier J. R. Rochell S. J. Dridi S. Sirri F. (2022). A review of heat stress in chickens. Part I: insights into physiology and gut health. Front. Physiol. 1535, 934381. 10.3389/fphys.2022.934381 PMC9386003 35991182

[B15] Butterfield D. A. Stadtman E. R. (1997). “Protein oxidation processes in aging brain,” in Advances in cell aging and gerontology. Editors Timiras P. S. Bittar E. E. (Stamford, CT: JAI Press), 2, 161–191. 10.1016/S1566-3124(08)60057-7

[B16] Cartoni Mancinelli A. Mattioli S. Dal Bosco A. Aliberti A. Guarino Amato M. Castellini C. (2020). Performance, behavior, and welfare status of six different organically reared poultry genotypes. Animals 10, 550. 10.3390/ani10040550 32218195 PMC7222370

[B17] Cartoni Mancinelli A. Mattioli S. Menchetti L. Dal Bosco A. Chiattelli D. Angelucci E. (2022). Validation of a behavior observation form for geese reared in agroforestry systems. Sci. Rep. 12, 15152–15213. 10.1038/s41598-022-18070-6 36071073 PMC9452672

[B18] Christie W. W. (1982). A simple procedure for rapid transmethylation of glycerolipids and cholesteryl esters. J. Lipid Res. 23, 1072–1075. 10.1016/S0022-2275(20)38081-0 6897259

[B19] CIE (1978). Recommendations on uniform color spaces, color differences, equations. Psychometric color terms. CIE 15, Suppl. 2. Paris: Commission Internationale de l’Eclairage.

[B20] Corwin D. L. (2021). Climate change impacts on soil salinity in agricultural areas. Eur. J. Soil Sci. 72, 842–862. 10.1111/ejss.13010

[B21] D'Souza D. N. Leury B. J. Dunshea F. R. Warner R. D. (1998). Effect of on-farm and pre-slaughter handling of pigs on meat quality. Aust. J. Agric. Res. 49, 1021–1025. 10.1071/A98010

[B22] Dai S. F. Gao F. Xu X. L. Zhang W. H. Song S. X. Zhou G. H. (2012). Effects of dietary glutamine and gamma-aminobutyric acid on meat colour, pH, composition, and water-holding characteristic in broilers under cyclic heat stress. Br. Poult. Sci. 53, 471–481. 10.1080/00071668.2012.719148 23130582

[B23] Dalle-Donne I. Rossi R. Giustarini D. Milzani A. Colombo R. (2003). Protein carbonyl groups as biomarkers of oxidative stress. Clin. Chim. Acta. 329, 23–38. 10.1016/S0009-8981(03)00003-2 12589963

[B24] Dias R. C. Krabbe E. L. Bavaresco C. Stefanello T. B. Kawski V. L. Panisson J. C. (2020). Effect of strain and nutritional density of the diet on the water-protein ratio, fat and collagen levels in the breast and legs of broilers slaughtered at different ages. Poult. Sci. 99, 2033–2040. 10.1016/j.psj.2019.11.033 32241488 PMC7587894

[B25] Elshafaei H. E. Rashed R. R. Goma A. A. El-kazaz S. E. Downing J. A. (2021). Performance, behaviour, breast yield and AME of meat chickens fed a reduced protein finisher diet while exposed to severe acute or moderate chronic thermal challenges. Livest. Sci. 251, 104669. 10.1016/j.livsci.2021.104669

[B26] European Commission (2010). Council Directive 2010/63/EU of 22 September 2010 on the protection of animals used for scientific purposes. Off. J. L 276, 33–79.

[B27] European Commission (2007). Council Directive (EC) No 43/2007 of 28 June 2007 laying down minimum rules for the protection of chickens kept for meat production. Off. J. L 182, 19–28.

[B28] European Commission (2009). Council Regulation (EC) No 1099/2009 of 24 September 2009 on the protection of animals at the time of killing. Off. J. L 303, 1–30.

[B29] FAO (2016). Climate change and food security: Risks and responses. Rome, Italy: Food and Agriculture Organization of the United Nations. Available at: http://www.fao.org/3/a-i5188e.pdf (Accessed on October 17, 2022).

[B30] Folch J. Lees M. Sloane Stanley G. H. (1957). A simple method for the isolation and purification of total lipides from animal tissues. J. Biol. Chem. 226, 497–509. 10.1016/s0021-9258(18)64849-5 13428781

[B31] Gonzalez-Rivas P. A. Chauhan S. S. Ha M. Fegan N. Dunshea F. R. Warner R. D. (2020). Effects of heat stress on animal physiology, metabolism, and meat quality: A review. Meat Sci. 162, 108025–108038. 10.1016/j.meatsci.2019.108025 31841730

[B32] Gregory N. G. (2010). How climatic changes could affect meat quality. Food Res. Int. 43, 1866–1873. 10.1016/j.foodres.2009.05.018

[B33] Hewavitharana A. K. Lanari M. C. Becu C. (2004). Simultaneous determination of vitamin E homologs in chicken meat by liquid chromatography with fluorescence detection. J. Chromatogr. A 1025, 313–317. 10.1016/j.chroma.2003.10.052 14763816

[B34] Hughes J. M. Oiseth S. K. Purslow P. P. Warner R. D. (2014). A structural approach to understanding the interactions between colour, water-holding capacity and tenderness. Meat Sci. 98, 520–532. 10.1016/j.meatsci.2014.05.022 25034451

[B35] Imik H. Ozlu H. Gumus R. E. C. E. P. Atasever M. A. Urcar S. Atasever M. (2012). Effects of ascorbic acid and α-lipoic acid on performance and meat quality of broilers subjected to heat stress. Br. Poult. Sci. 53, 800–808. 10.1080/00071668.2012.740615 23398425

[B36] Jeacocke R. E. (1977). Continuous measurements of the pH of beef muscle in intact beef carcases. Int. J. Food Sci. Technol. 12, 375–386. 10.1111/j.1365-2621.1977.tb00120.x

[B37] Jiang Q. Elson-Schwab I. Courtemanche C. Ames B. N. (2000). gamma-tocopherol and its major metabolite, in contrast to alpha-tocopherol, inhibit cyclooxygenase activity in macrophages and epithelial cells. Proc. Natl. Acad. Sci. U. S. A. 97, 11494–11499. 10.1073/pnas.200357097 11005841 PMC17228

[B38] Kadim I. T. Mahgoub O. Al-Marzooqi W. Al-Ajmi D. S. Al-Maqbali R. S. Al-Lawati S. M. (2008). The influence of seasonal temperatures on meat quality characteristics of hot-boned, m. psoas major and minor, from goats and sheep. Meat Sci. 80, 210–215. 10.1016/j.meatsci.2007.11.022 22063324

[B39] Kikusato M. Toyomizu M. (2019). Differential effects of heat stress on oxidative status of skeletal muscle with different muscle fibre compositions in broiler chicken. J. Anim. Feed Sci. 28, 78–82. 10.22358/jafs/102830/2019

[B40] Kim Y. H. B. Warner R. D. Rosenvold K. (2014). Influence of high pre-rigor temperature and fast pH fall on muscle proteins and meat quality: A review. Anim. Prod. Sci. 54, 375–395. 10.1071/AN13329

[B41] Kumar M. Ratwan P. Dahiya S. P. Nehra A. K. (2021). Climate change and heat stress: impact on production, reproduction and growth performance of poultry and its mitigation using genetic strategies. J. Therm. Biol. 97, 102867. 10.1016/j.jtherbio.2021.102867 33863431

[B42] Lara L. J. Rostagno M. H. (2013). Impact of heat stress on poultry production. Animals 3, 356–369. 10.3390/ani3020356 26487407 PMC4494392

[B43] Leishman E. M. Ellis J. van Staaveren N. Barbut S. Vanderhout R. J. Osborne V. R. (2021). Meta-analysis to predict the effects of temperature stress on meat quality of poultry. Poult. Sci. 100, 101471. 10.1016/j.psj.2021.101471 34607155 PMC8496168

[B44] Liu L. Ren M. Ren K. Jin Y. Yan M. (2020). Heat stress impacts on broiler performance: A systematic review and meta-analysis. Poult. Sci. 99, 6205–6211. 10.1016/j.psj.2020.08.019 33142538 PMC7647856

[B45] Lu Z. He X. F. Ma B. B. Zhang L. Li J. L. Jiang Y. (2019). Increased fat synthesis and limited apolipoprotein B cause lipid accumulation in the liver of broiler chickens exposed to chronic heat stress. Poult. Sci. 98, 3695–3704. 10.3382/ps/pez056 30809677

[B46] Lu Z. He X. Ma B. Zhang L. Li J. Jiang Y. (2017). Chronic heat stress impairs the quality of breast-muscle meat in broilers by affecting redox status and energy-substance metabolism. J. Agric. Food Chem. 65, 11251–11258. 10.1021/acs.jafc.7b04428 29212325

[B47] Lu Z. He X. Ma B. Zhang L. Li J. Jiang Y. (2018). Serum metabolomics study of nutrient metabolic variations in chronic heat-stressed broilers. Br. J. Nutr. 119, 771–781. 10.1017/S0007114518000247 29569538

[B48] Ma B. He X. Lu Z. Zhang L. Li J. Jiang Y. (2018). Chronic heat stress affects muscle hypertrophy, muscle protein synthesis and uptake of amino acid in broilers via insulin like growth factor-mammalian target of rapamycin signal pathway. Poult. Sci. 97, 4150–4158. 10.3382/ps/pey291 29982693

[B49] Mack L. A. Felver-Gant J. N. Dennis R. L. Cheng H. W. (2013). Genetic variations alter production and behavioral responses following heat stress in 2 strains of laying hens. Poult. Sci. 92, 285–294. 10.3382/ps.2012-02589 23300291

[B50] Marìa G. A. Escós J. Alados C. L. (2004). Complexity of behavioural sequences and their relation to stress conditions in chickens (Gallus gallus domesticus): A non-invasive technique to evaluate animal welfare. Appl. Anim. Behav. Sci. 86, 93–104. 10.1016/j.applanim.2003.11.012

[B51] Mattioli S. Dal Bosco A. Duarte J. M. M. D’Amato R. Castellini C. Beone G. M. (2019). Use of selenium-enriched olive leaves in the feed of growing rabbits: effect on oxidative status, mineral profile and selenium speciation of longissimus dorsi meat. J. Trace Elem. Med. Biol. 51, 98–105. 10.1016/j.jtemb.2018.10.004 30466946

[B52] Mattioli S. Mancinelli A. C. Menchetti L. Dal Bosco A. Madeo L. Guarino Amato M. (2021). How the kinetic behavior of organic chickens affects productive performance and blood and meat oxidative status: A study of six poultry genotypes. Poult. Sci. 100, 101297. 10.1016/j.psj.2021.101297 34280645 PMC8319010

[B53] Mazzoni M. Zampiga M. Clavenzani P. Lattanzio G. Tagliavia C. Sirri F. (2022). Effect of chronic heat stress on gastrointestinal histology and expression of feed intake-regulatory hormones in broiler chickens. Animal 16, 100600. 10.1016/j.animal.2022.100600 35907384

[B54] Pawar S. S. Sajjanar B. Lonkar V. D. Kurade N. P. Kadam A. S. Nirmal A. V. (2016). Assessing and mitigating the impact of heat stress in poultry. Adv. Anim. Vet. Sci. 4, 332–341. 10.14737/journal.aavs/2016/4.6.332.341

[B55] International Panel on Climate Change (2022). “Summary for policymakers,” in Climate change 2022: Impacts, adaptation and vulnerability. Contribution of working group II to the sixth assessment report of the intergovernmental Panel on climate change. Editors Pörtner H.-O. Roberts D. C. Poloczanska E. S. Mintenbeck K. Tignor M. Alegría A. (Cambridge: Cambridge University Press), 3–33. 10.1017/9781009325844.001

[B56] Rhoads R. P. La Noce A. J. Wheelock J. B. Baumgard L. H. (2011). Alterations in expression of gluconeogenic genes during heat stress and exogenous bovine somatotropin administration. Int. J. Dairy Sci. 94, 1917–1921. 10.3168/jds.2010-3722 21426982

[B57] Ricciotti E. FitzGerald G. A. (2011). Prostaglandins and inflammation. Arterioscler. Thromb. Vasc. Biol. 31, 986–1000. 10.1161/ATVBAHA.110.207449 21508345 PMC3081099

[B58] Schreurs F. J. G. (2000). Post-mortem changes in chicken muscle: some key biochemical processes involved in the conversion of muscle to meat. World Poult. Sci. J. 56, 319–346. 10.1079/wps20000023

[B59] Schüep W. Rettenmaier R. (1994). Analysis of vitamin E homologs in plasma and tissue: high-performance liquid chromatography. Methods Enzymol. 234, 294–302. 10.1016/0076-6879(94)34096-X 7808296

[B60] Shakeri M. Cottrell J. J. Wilkinson S. Le H. H. Suleria H. A. Warner R. D. (2020). Dietary betaine reduces the negative effects of cyclic heat exposure on growth performance, blood gas status and meat quality in broiler chickens. Agriculture 10, 176–188. 10.3390/agriculture10050176 PMC7401608 32650461

[B61] Shakeri M. Le H. H. (2022). Deleterious effects of heat stress on poultry production: unveiling the benefits of betaine and polyphenols. Poultry 1, 147–156. 10.3390/poultry1030013

[B62] Soglia F. Petracci M. Ertbjerg P. (2016). Novel DNPH-based method for determination of protein carbonylation in muscle and meat. Food Chem. 197, 670–675. 10.1016/j.foodchem.2015.11.038 26617002

[B64] Syafwan S. Kwakkel R. P. Verstegen M. W. A. (2011). Heat stress and feeding strategies in meat-type chickens. World Poult. Sci. J. 67, 653–674. 10.1017/S0043933911000742

[B65] Sykes A. H. Fataftah A. R. A. (1986). Acclimatization of the fowl to intermittent acute heat stress. Br. Poult. Sci. 27, 289–300. 10.1080/00071668608416881 3742264

[B66] Tabiri H. Y. Sato K. Takahashi K. Toyomizu M. Akiba Y. (2002). Effects of heat stress and dietary tryptophan on performance and plasma amino acid concentrations of broiler chickens. Asian Australas. J. Anim. Sci. 15, 247–253. 10.5713/ajas.2002.247

[B67] Talebi E. Dolatkhah A. Joyani M. (2022). The effect of high temperature on poultry and effective factors on reducing the adverse effects of heat stress: A review. J. Emerg. Trends Eng. Appl. Sci. 13, 38–43. https://hdl.handle.net/10520/ejc-sl_jeteas_v13_n3_a2 .

[B68] Van Laack R. L. J. M. Liu C. H. Smith M. O. Loveday H. D. (2000). Characteristics of pale, soft, exudative broiler breast meat. Poult. Sci. 79, 1057–1061. 10.1093/ps/79.7.1057 10901210

[B69] Verma U. Gautam M. Parmar B. Khaire K. Wishart D. S. Balakrishnan S. (2021). New insights into the obligatory nature of cyclooxygenase-2 and PGE2 during early chick embryogenesis. Biochim. Biophys. Acta - Mol. Cell Biol. Lipids 1866, 158889. 10.1016/j.bbalip.2021.158889 33454433

[B70] Wang R. H. Liang R. R. Lin H. Zhu L. X. Zhang Y. M. Mao Y. W. (2017). Effect of acute heat stress and slaughter processing on poultry meat quality and postmortem carbohydrate metabolism. Poult. Sci. 96, 738–746. 10.3382/ps/pew329 27702924

[B71] Warriss P. D. (2000). Meat science: An introductory text. New York: CABI Publishing. 10.1016/s0309-1740(00)00053-x 22062084

[B72] Wu W. Zhang C. Hua Y. (2009). Structural modification of soy protein by the lipid peroxidation product acrolein. J. Sci. Food. Agric. 89, 133–140. 10.1016/j.lwt.2009.05.006

[B73] Xing T. Gao F. Tume R. K. Zhou G. Xu X. (2019). Stress effects on meat quality: A mechanistic perspective. Compr. Rev. Food Sci. 18, 380–401. 10.1111/1541-4337.12417 33336942

[B74] Yahav S. Goldfeld S. Plavnik I. Hurwitz S. (1995). Physiological responses of chickens and turkeys to relative humidity during exposure to high ambient temperature. J. Therm. Biol. 3, 245–253. 10.1016/0306-4565(94)00046-L

[B75] Zaboli G. Huang X. Feng X. Ahn D. U. (2019). How can heat stress affect chicken meat quality? a review. Poult. Sci. 98, 1551–1556. 10.3382/ps/pey399 30169735

[B76] Zampiga M. Flees J. Meluzzi A. Dridi S. Sirri F. (2018). Application of omics technologies for a deeper insight into quali-quantitative production traits in broiler chickens: A review. J. Anim. Sci. Biotechnol. 9, 61–18. 10.1186/s40104-018-0278-5 30214720 PMC6130060

[B77] Zampiga M. Laghi L. Zhu C. Cartoni Mancinelli A. Mattioli S. Sirri F. (2021). Breast muscle and plasma metabolomics profile of broiler chickens exposed to chronic heat stress conditions. Animal 15, 100275. 10.1016/j.animal.2021.100275 34120075

[B78] Zampiga M. Soglia F. Baldi G. Petracci M. Strasburg G. M. Sirri F. (2020). Muscle abnormalities and meat quality consequences in modern Turkey hybrids. Front. Physiol. 11, 554. 10.3389/fphys.2020.00554 32595515 PMC7304436

[B79] Zeferino C. P. Komiyama C. M. Pelícia V. C. Fascina V. B. Aoyagi M. M. Coutinho L. L. (2016). Carcass and meat quality traits of chickens fed diets concurrently supplemented with vitamins C and E under constant heat stress. Animal 10, 163–171. 10.1017/S1751731115001998 26677935

[B80] Zhao J. Chen J. Zhu H. Xiong Y. L. (2012). Mass spectrometric evidence of malonaldehyde and 4-hydroxynonenal adductions to radical-scavenging soy peptides. J. Agric. Food Chem. 60, 9727–9736. 10.1021/jf3026277 22946674 PMC3580276

